# Response-locked theta dissociations reveal potential feedback signal following successful retrieval

**DOI:** 10.1162/imag_a_00207

**Published:** 2024-06-27

**Authors:** Devyn E. Smith, Justin R. Wheelock, Nicole M. Long

**Affiliations:** Department of Psychology, University of Virginia, Charlottesville, VA, United States

**Keywords:** theta, EEG, episodic memory, retrieval

## Abstract

Successful memory retrieval relies on memory processes to access an internal representation and decision processes to evaluate and respond to the accessed representation, both of which are supported by fluctuations in theta (4–8 Hz) activity. However, the extent to which decision-making processes are engaged following a memory response is unclear. Here, we recorded scalp electroencephalography (EEG) while human participants performed a recognition memory task. We focused on response-locked data, allowing us to investigate the processes that occur prior to and following a memory response. We replicate previous work and find that prior to a memory response theta power is greater for identification of previously studied items (hits) relative to rejection of novel lures (correct rejections; CRs). Following the memory response, the theta power dissociation “flips” whereby theta power is greater for CRs relative to hits. We find that the post-response “flip” is more robust for hits that are committed quickly, potentially reflecting a positive feedback signal for strongly remembered experiences. Our findings suggest that there are potentially distinct processes occurring before and after a memory response that are modulated by successful memory retrieval.

## 1 Introduction

Successful remembering is dependent on both memory processes to access stored representations and decision processes to evaluate and respond to the accessed representation. How these processes unfold over time, and the underlying neural mechanisms, are critically important to memory success, yet our understanding of these processes remains limited. In particular, convergent evidence from scalp electroencephalography (EEG) studies in both the memory literature (Klimesch et al., 1997; Nyhus & Curran, 2010) and the decision-making literature (Frank et al., 2015; Pinner & Cavanagh, 2017; Senftleben & Scherbaum, 2021) suggest that frontocentral theta (4–8 Hz activity in EEG) supports both memory and decision-making processes. However, the extent to which theta dissociations in a memory task reflect decision-making processes is unclear, likely due to limited investigation of EEG signals *following* a memory response. The aim of this study is to investigate the neural correlates leading up to and following a memory response.

It is well established that successful remembering is characterized by electrophysiological changes around 300 to 800 ms following stimulus onset during a memory test (Addante et al., 2012; Friedman & Johnson Jr., 2000; Rugg & Wilding, 2000; Voss & Paller, 2008). Specifically, two event-related potentials (ERPs) distinguish successful remembering of a target or studied item (hit) from successful rejection of a lure or non-studied item (correct rejection, CR). The FN400, a negative going frontal ERP component thought to reflect familiarity (Curran & Hancock, 2007; Mecklinger, 2000; Rugg et al., 1998), is more negative for CRs than hits around 300 to 500 ms after stimulus onset (Curran, 2000, 2004; Curran & Cleary, 2003) and the LPC, a late positive going parietal ERP component thought to reflect recollection (Friedman & Johnson Jr., 2000; Mecklinger, 2000), is more positive for hits than CRs around 400 to 800 ms after stimulus onset (Curran, 2000, 2004; Curran & Cleary, 2003; Friedman & Johnson Jr., 2000). Similarly, theta power is greater for hits compared to CRs, most often around 500 to 1000 ms after stimulus onset (Burgess & Gruzelier, 1997; Düzel et al., 2003; Klimesch et al., 2000). According to the drift diffusion model, recognition memory is supported by a mechanism whereby evidence accumulates over time until a threshold is reached and a decision is made (Ratcliff & McKoon, 2008). Given evidence that theta power is positively correlated with reaction times, such that theta power increases until a response is made (Jacobs et al., 2006), theta power prior to a response may reflect evidence accumulation and/or reinstatement (Guan et al., 2023; Herweg et al., 2020; Kota et al., 2020; Nyhus & Curran, 2010). However, these effects are related to representation access prior to a response and as such, do not elucidate the processes that may unfold after a memory response is made.

Parallel findings from the decision-making and cognitive control literature have revealed that both ERPs and theta power track errors and negative feedback signals prior to and following a response (Cavanagh & Frank, 2014; Cavanagh et al., 2010; Luft, 2014; Luu et al., 2004; Trujillo & Allen, 2007). Specifically, the error-related negativity (ERN), a negative going fronto central ERP component that reflects decision conflict or error monitoring (Frank et al., 2005; L. Wang et al., 2020), is more negative following incorrect compared to correct responses (Cavanagh et al., 2012; Gehring et al., 1993). Similarly, across cognitive control tasks such as the Stroop task, the flanker task, and go/no-go tasks, theta power is greater following incorrect compared to correct responses and following negative relative to positive outcomes (Cavanagh & Frank, 2014; Cohen, 2014b; Mazaheri et al., 2009). Together, these findings suggest that post-response theta power may reflect a feedback signal or monitoring process.

Although there is evidence for post-retrieval monitoring during recognition memory (Hill et al., 2021; Rugg et al., 2003), these signals are often measured following access of the representation, but prior to the memory response itself. Further complicating interpretation is that reaction times are generally faster for hits than CRs (Uncapher et al., 2015; Weidemann & Kahana, 2016), meaning that stimulus-locked hit versus CR dissociations may include both pre- and post-response related processes. That is, insofar as an evaluative or updating decision-making process is engaged after a memory response has been made, stimulus-locked comparisons may contrast post-hit evaluative decision-making processing with pre-CR memory retrieval-related processing. There is ERP evidence that post-retrieval monitoring processes are supported by a late old/new effect over right frontal cortex (Johansson & Mecklinger, 2003; Wilding & Rugg, 1996), characterized by a positive voltage deflection that is greater for hits than CRs (Hayama et al., 2008). However, the majority of such post-retrieval monitoring signals occur after the putative representation has been accessed, but before a behavioral response is made (Cruse & Wilding, 2009, 2011; Woodruff et al., 2006), leaving open the question of whether decision-making mechanisms are engaged following a memory response. Greater conflict between memory decisions in a recognition task—created via differential payoff rates for correct old versus new responses—leads to a greater post-response ERN (Curran, DeBuse, et al., 2007), suggesting that control or monitoring processes may be engaged after a memory response is made.

Our hypothesis is that distinct processes occur prior to and following a memory response. To test our hypothesis, we conducted a human scalp EEG recognition memory study in which we specifically assessed response-locked theta power and provided no explicit feedback to participants. By investigating response-locked signals, we can separately assess pre- and post-response related processing during both hits and CRs. We expected to replicate prior work and find greater theta power for hits compared to CRs preceding the response (equivalent to the established stimulus-locked effects, e.g., Burgess & Gruzelier, 1997; Düzel et al., 2003; Nyhus & Curran, 2010). To the extent that distinct, and potentially decision-making-related, processes are engaged following a memory response, we expected to find a post-response theta pattern that differed from the pre-response hit versus CR effect. First, if there are neither memory nor decision-making-related signals following a memory response, theta power following both hits and CRs should return to baseline. Alternatively, because both hits and CRs are correct responses, theta power may be similarly decreased for both response types following a memory response. Finally, given that the reward system—in particular, the striatum—has previously been linked to successful memory retrieval, whereby the striatum shows greater activity for hits compared to CRs (Clos et al., 2015; Spaniol et al., 2009), successful retrieval may be intrinsically rewarding (Satterthwaite et al., 2012; Speer et al., 2014). Therefore, theta power may dissociate post-response hits from CRs such that theta power would be greater for CRs compared to hits following the memory response, reflecting a positive feedback signal for hits. The direct comparison of two classes of responses that are both accurate, but differ in terms of successful retrieval, enables adjudication between these alternative hypotheses.

## 2 Materials and Methods

### 2.1 Participants

Forty (30 female; age range = 18–42, mean age = 21.9 years) native English speakers from the University of Virginia community participated. Our sample size of N = 40 was selected based on prior scalp EEG studies conducted in our lab (Moore & Long, 2024; Smith et al., 2022). All participants had normal or corrected-to-normal vision. Informed consent was obtained in accordance with University of Virginia Institutional Review Board for Social and Behavioral Research, and participants were compensated for their participation. Two participants were excluded from the final dataset: one for technical difficulties that resulted in a subset of test items being presented twice during the test phase and one who failed to comply with task instructions. Thus, data are reported for the remaining 38 participants. All raw, de-identified data and the associated experimental and analysis codes used in this study can be accessed via the Long Term Memory Lab Website (https://longtermmemorylab.com).

### 2.2 Recognition task experimental design

Stimuli consisted of 1602 words, drawn from the Toronto Noun Pool (Friendly et al., 1982). From this set, 288 words were randomly selected for each participant. Of these words, 192 were presented in both the study and test phase while the remaining 96 served as lures in the test phase.

#### 2.2.1 Study phase

**Fig. 1.**
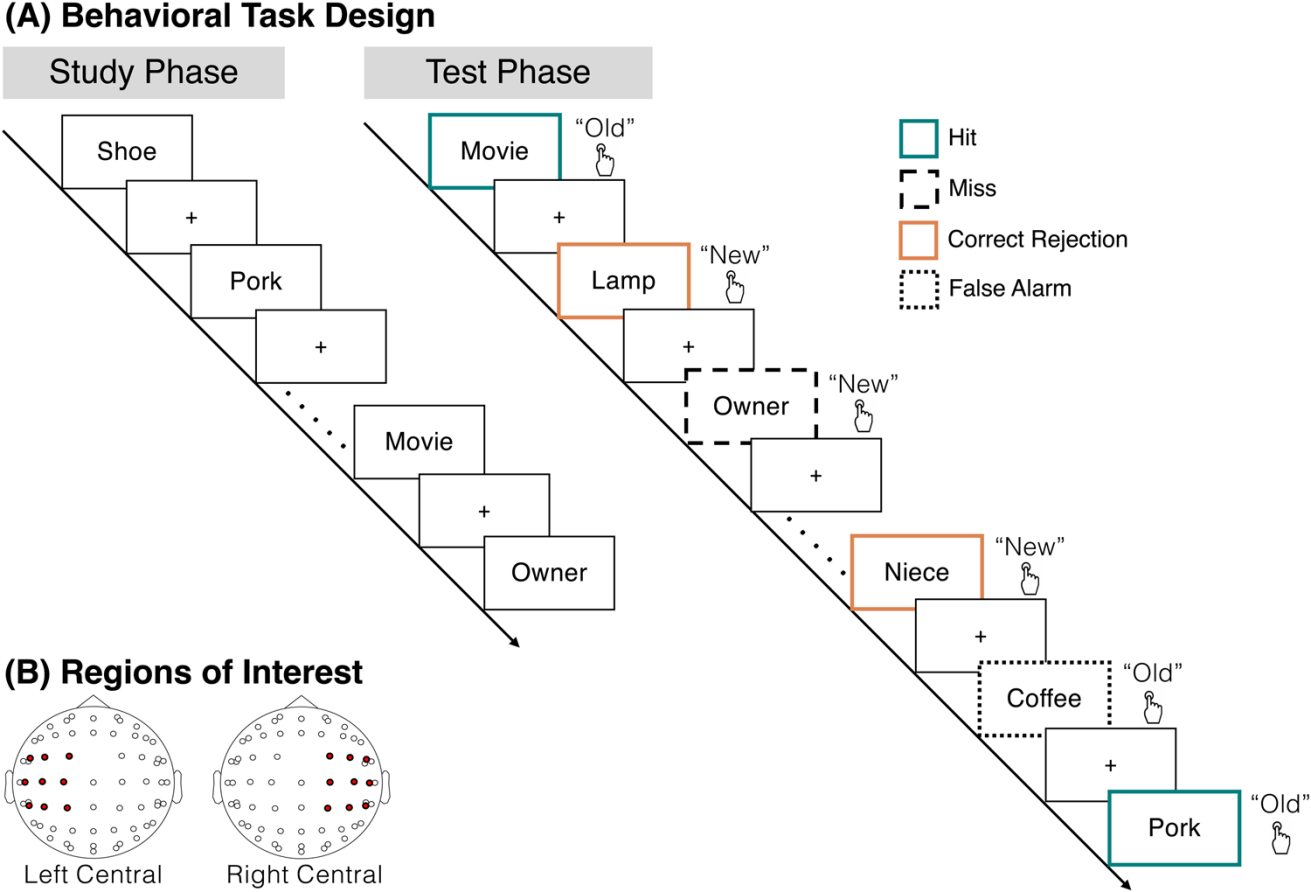
Task design. (A) During the study phase, participants studied individual words in anticipation of a later memory test and made no behavioral responses. After 12 runs of 16-item word lists, participants completed a recognition test phase. On each trial, participants saw either a target, a word that was presented during the study phase, or a lure, a word that was not presented during the study phase. The participants’ task was to make an old or new judgment for each word. There were one of four possible response types, hits (teal; an “old” response to a target), correct rejections (orange; a “new” response to a lure), misses (dashed black lines; a “new” response to a target), and false alarms (dotted black lines; an “old” response to a lure). Lines and colors around the boxes are shown for illustrative purposes and were not present during the actual experiment. (B) We analyzed two regions of interest (ROIs), a left central ROI (FC5, FC1, C3, CP5, CP1, FC3, C1, C5, CP3) and a right central ROI (CP6, CP2, C4, FC6, FC2, CP4, C6, C2, FC4).

In each of 12 runs, participants viewed a list containing 16 words, yielding a total of 192 trials. During each trial, participants saw a single word presented for 2000 ms followed by a 1000 ms inter-stimulus interval (ISI; Fig. 1A). As we did not want to bias participants to the semantic features of the study items (Moore & Long, 2024), we did not include an encoding task. Instead, participants were instructed to study the presented word in anticipation for a later memory test and did not make any behavioral responses. An earlier motivation of this work was to investigate how semantic associations among study items influence memory formation mechanisms. To that end, each list was split evenly into two parts containing 8 words (“first associates” and “second associates,” respectively) separated by a brief 2000 ms delay. Semantic association strength was determined using Word Association Space values (WAS; Nelson et al., 2001); “strong” semantic associates had a WAS value of 0.4 or greater, and “weak” semantic associates had a WAS value less than 0.4 (Long & Kahana, 2017). Half of the first and second associates were strongly semantically associated, and half of the first and second associates were weakly semantically associated. Both strong and weak semantic associates were weakly semantically associated to all other study words. Word lists were generated for each participant by randomly drawing a word from the pool of 1602 words and selecting either a strong or weak associate from the word pool and then removing the selected word, selected associate, and all other strong semantic associates of the selected word, from the pool. We iteratively repeated this process until a total of 192 words were selected.

#### 2.2.2 Test phase

Following the 12 study runs, participants completed the recognition test phase. On each trial, participants viewed a word which had either been presented during the study phase (target) or had not been presented (lure; Fig. 1A). Participants’ task was to make an old or new judgment for each word by pressing one of two buttons (“d” or “k”). Response mappings were counterbalanced across participants. Test trials were self-paced, and responses could occur anytime after the stimulus onset. Participants received no feedback on the accuracy of their responses. Test trials were separated by a 1000 ms ISI. There were a total of 288 test trials with all 192 study words presented along with 96 novel lures, half of which were semantically associated to a study word. As we find similar effects when accounting for semantic associations (see Results), we collapsed analyses across strong and weak semantic associates.

### 2.3 EEG data acquisition and preprocessing

All acquisition and preprocessing methods are based on our previous work (Smith et al., 2022); for clarity we use the same text as previously reported. EEG recordings were collected using a BrainVision system and an ActiCap equipped with 64 Ag/AgCl active electrodes positioned according to the extended 10–20 system. All electrodes were digitized at a sampling rate of 1000 Hz and were referenced to electrode FCz. Offline, electrodes were later converted to an average reference. Impedances of all electrodes were kept below 50 kΩ. Electrodes that demonstrated high impedance or poor contact with the scalp were excluded from the average reference. Bad electrodes were determined by voltage thresholding (see below).

Custom python codes were used to process the EEG data. We applied a high-pass filter at 0.1 Hz, followed by a notch filter at 60 Hz and harmonics of 60 Hz to each participant’s raw EEG data. We then performed three preprocessing steps (Nolan et al., 2010) to identify electrodes with severe artifacts. First, we calculated the mean correlation between each electrode and all other electrodes as electrodes should be moderately correlated with other electrodes due to volume conduction. We z-scored these means across electrodes and rejected electrodes with z-scores less than -3. Second, we calculated the variance for each electrode, as electrodes with very high or low variance across a session are likely dominated by noise or have poor contact with the scalp. We then z-scored variance across electrodes and rejected electrodes with a |z| > = 3. Finally, we expect many electrical signals to be autocorrelated, but signals generated by the brain versus noise are likely to have different forms of autocorrelation. Therefore, we calculated the Hurst exponent, a measure of long-range autocorrelation, for each electrode and rejected electrodes with a |z| > = 3. Electrodes marked as bad by this procedure were excluded from the average re-reference. We then calculated the average voltage across all remaining electrodes at each time sample and re-referenced the data by subtracting the average voltage from the filtered EEG data. We used wavelet-enhanced independent component analysis (Castellanos & Makarov, 2006) to remove artifacts from eyeblinks and saccades.

### 2.4 EEG data analysis

We applied the Morlet wavelet transform (wave number 6) to the entire EEG time series across electrodes, for each of 46 logarithmically spaced frequencies (2–100 Hz; Long & Kahana, 2015). Because we hypothesized distinct processes occur prior to and following a memory response, after log-transforming the power we focused exclusively on test-phase data. We then downsampled the test-phase data by taking a moving average across 100 ms time intervals from -1000 to 3000 ms relative to the response and sliding the window every 25 ms, resulting in 157 time intervals (40 non-overlapping). Mean and standard deviation power were calculated across all trials and across time points for each frequency. Power values were then z-transformed by subtracting the mean and dividing by the standard deviation power. We focus exclusively on the theta band (4–8 Hz) for all analyses.

### 2.5 Regions of interest

We examined theta power across two regions of interest (ROIs; Fig. 1B): left central (FC5, FC1, C3, CP5, CP1, FC3, C1, C5, CP3) and right central (CP6, CP2, C4, FC6, FC2, CP4, C6, C2, FC4). We specifically focus on the frontocentral region as prior work has demonstrated that theta power in this region dissociates both hits and correct rejections (Burgess & Gruzelier, 1997; Gruber et al., 2008; Klimesch et al., 1997; Nyhus & Curran, 2010) and positive and negative feedback (Cohen et al., 2007; Marco-Pallarés et al., 2008; Mas-Herrero et al., 2015).

### 2.6 Univariate analyses

To test the effect of response type on theta power leading up to and following memory responses, our two conditions of interest were hits (correctly recognized targets) and correct rejections (CRs, correctly rejected lures). We compared theta power across hits and CRs separately for each ROI. For each participant, we calculated z-transformed theta power across both ROIs in each of the two conditions, across 100 ms time intervals from 500 ms pre-response to 1000 ms post-response. For a direct comparison of pre-response and post-response theta power, we further averaged z-transformed theta power over the 500 ms pre-response and 500 ms post-response interval separately for hits and CRs. We selected 500 ms as our pre-response interval based on our prior work investigating contextually mediated retrieval processes in the hippocampus (Long et al., 2017).

### 2.7 Peak analysis

To measure the center frequency (CF) of theta leading up to and following memory responses, we used fitting oscillations and one over f (FOOOF; Ostlund et al., 2022). To specifically measure periodic signals, for each participant, we fit the FOOOF model to every test trial and extracted all identified CFs within the theta band prior to and following the response for both ROIs. We compared the averaged CF over the 500 ms pre-response and 500 ms post-response interval between hits and CRs.

### 2.8 Statistical analyses

We used a repeated-measures ANOVA (rmANOVA) to assess the distribution of reaction times (RTs) for hits and CRs. For post hoc comparisons across RTs, we used false discovery rate (FDR; *p* = 0.05) correction (Benjamini & Hochberg, 1995) to correct for multiple comparisons. We used rmANOVAs and paired-sample *t*-tests to assess the effect of response type (hits, CRs) and time interval (pre-response, post-response) on theta power.

## 3 Results

**Fig. 2.**
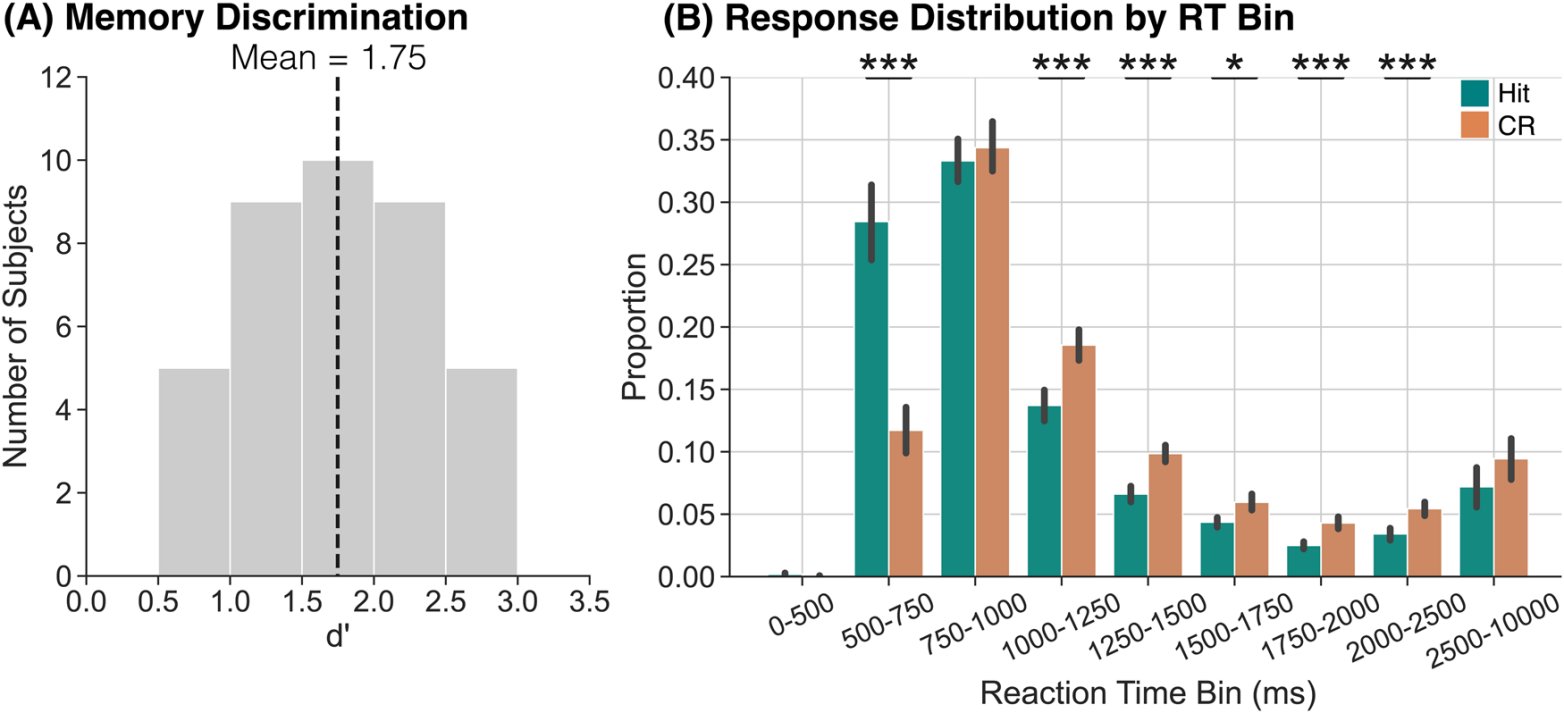
Memory discrimination and responses as a function of reaction time bin. (A) We used d’ to assess memory discrimination. Participants were able to correctly discriminate between targets and lures. (B) We assessed the proportion of hits (teal) and CRs (orange) as a function of RT bin. The highest proportion of hits and CRs occurs in the 750–1000 ms bin, and a significantly greater proportion of hits compared to CRs occur within the 500–750 ms bin. Error bars reflect standard error of the mean. *p < 0.05; ***p < 0.001 (FDR-corrected).

Our first goal was to measure memory discrimination (d’) to ensure that participants were following directions and able to discriminate between targets and lures. For each participant, we calculated d’ by subtracting the normalized false alarm rate (the percentage of lures that were incorrectly identified as “old”) from the normalized hit rate (the percentage of targets that were correctly identified as “old”). The average d’ was 1.75 (SD = 0.58; Fig. 2A), indicating that participants were able to successfully distinguish targets from lures.

Having found that participants are able to discriminate targets and lures, we next assessed median reaction times (RTs) for hits and CRs. To the extent that RTs reliably differ between hits and CRs, stimulus-locked neural dissociations between these conditions may be driven by engagement of different processes, for example, memory versus decision-making, rather than differential engagement of the same process. That is, if hits occur more quickly than CRs, a stimulus-locked comparison between the two conditions could reflect a comparison between post-response processes for hits versus pre-response processes for CRs. The average median RT for hits (M = 954.9, SD = 419.6) was significantly faster than for CRs (M = 1122.8, SD = 317.3, *t*_37_ = -4.427, *p* = 0.0001, *d* = 0.4514). This RT difference suggests that stimulus-locked theta dissociations could be driven by dissociations in pre- versus post-response processes across hits and CRs.

Given the dissociation in median RT between hits and CRs, we next sought to compare the distribution of RTs across conditions to determine when relative to stimulus onset the majority of hits and CRs occur. We grouped RTs into nine bins selected to cover the full range of RTs with higher resolution in the faster (<2500 ms) bins: 0–500 ms, 500–750 ms, 750–1000 ms, 1000–1250 ms, 1250–1500 ms, 1500–1750 ms, 1750–2000 ms, 2000–2500 ms, and 2500–10000 ms (Fig. 2B). We calculated the proportion of hits and CRs that occurred within each RT bin for each participant and then averaged those proportions across all participants. We conducted a 2 × 9 rmANOVA with factors of response type (hit, CR) and RT bin. We do not find a significant main effect of response type (*F*_1,37_ = 0.51, *p* = 0.479, $\eta_{p}^{2}$ = 0.01). We find a significant main effect of RT bin (*F*_8,296_ = 74.48, *p* < 0.0001, $\eta_{p}^{2}$ = 0.67) and a significant interaction between response type and RT bin (*F*_8,296_ = 22.36, *p* < 0.0001, $\eta_{p}^{2}$ = 0.38). We report the results of post hoc *t*-tests comparing proportions of hits and CRs within each RT bin in Table 1 and highlight the key findings below. We find that the highest proportion of responses occurs in the 750–1000 ms RT bin for both hits and CRs. However, a significantly greater proportion of hits (M = 0.28, SD = 0.19) occur within the 500–750 ms RT bin compared to CRs (M = 0.12, SD = 0.11). Thus, if participants evaluate or update a representation after responding, neural activity observed within the 500–750 ms interval may reflect a comparison of post-hit evaluation processes with pre-CR memory or retrieval-related processes.

**Table 1.** Post hoc *t*-tests comparing the proportion of hits and CRs in each RT bin.

	Hits		CRs		Hits vs. CRs		
RT bin (ms)	Mean	SD	Mean	SD	$t_{37}$	*p*	*d*
0–500	0.002	0.006	0.0004	0.002	1.579	0.1229	0.3252
500–750	0.28	0.19	0.12	0.11	7.058	<**0.0001**	1.081
750–1000	0.33	0.11	0.34	0.13	-0.466	0.6438	0.0898
1000–1250	0.14	0.08	0.19	0.07	-4.198	**0.0002**	0.6536
1250–1500	0.07	0.04	0.10	0.04	-4.425	**0.0001**	0.7708
1500–1750	0.04	0.02	0.06	0.04	-2.549	**0.02**	0.4681
1750–2000	0.02	0.02	0.04	0.03	-4.012	**0.0003**	0.7581
2000–2500	0.03	0.03	0.05	0.03	-3.973	**0.0003**	0.6373
2500–10000	0.07	0.10	0.09	0.10	-2.009	0.052	0.2192

*Note*: Bold values indicate tests that survive FDR correction.

Our hypothesis is that distinct memory and decision-making processes occur preceding and following a memory response, meaning that we should find differential theta power engagement pre- and post-response. Specifically, we should replicate past findings of greater theta power for hits compared to CRs pre-response, reflecting memory-related processing. To the extent that decision-making processes are engaged following a response, we should either find decreased theta power for both hits and CRs—as both conditions are correct responses—or we may find a theta dissociation between hits and CRs. That is, to the extent that successful retrieval is intrinsically rewarding, post-response theta power should be decreased for hits compared to CRs. We conducted a 2 × 2 × 15 rmANOVA with factors of response type (hit, CR), ROI (left central; LC, right central; RC) and time interval (-500 to 1000 ms in fifteen 100 ms intervals). We report the results of this ANOVA in Table 2 and highlight the key findings here. We find a significant interaction between response type and time interval (*F*_14,518_ = 6.127, *p* < 0.0001, $\eta_{p}^{2}$ = 0.14) which indicates that theta power dissociations between hits and CRs changes over time.

**Table 2.** Analysis of variance results for response type (hit, CR), ROI, and time interval (-500 to 1000 ms in fifteen 100 ms intervals) on theta power.

Effect	*df*	*F*	*p*	$\eta_{p}^{2}$
Main effect of response type	(1,37)	0.172	0.681	0.005
Main effect of ROI	(1,37)	11.36	**0.002**	0.23
Main effect of time interval	(14,518)	21.59	<**0.0001**	0.37
Interaction of response type × ROI	(1,37)	0.218	0.643	0.006
Interaction of ROI × time interval	(14,518)	3.408	<**0.0001**	0.08
Interaction of response type × time interval	(14,518)	6.127	<**0.0001**	0.14
Interaction of response type × ROI × time interval	(14,518)	2.23	**0.006**	0.06

*Note*: Bold values indicate *p* < 0.05.

**Fig. 3.**
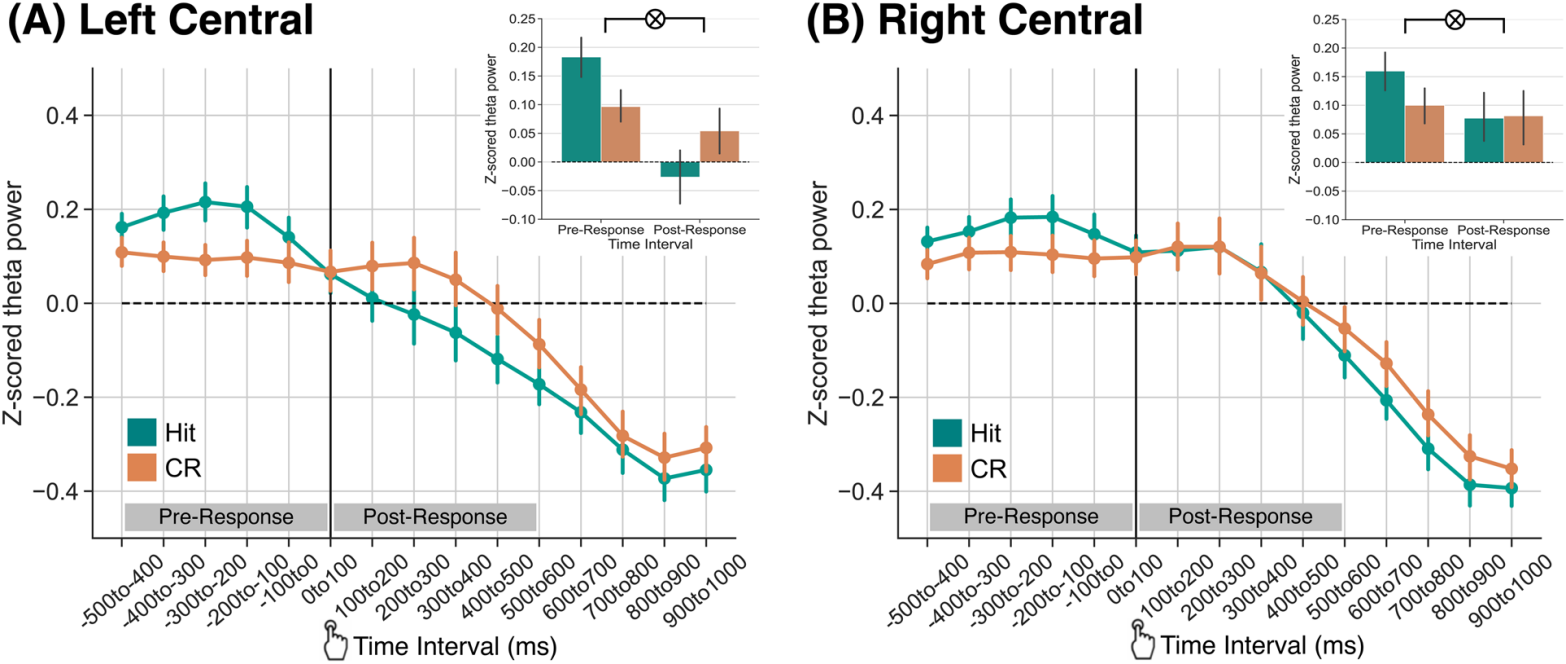
Theta power dissociations across hits and correct rejections preceding and following memory responses. Response-locked z-transformed theta power (4–8 Hz) for the left and right central ROIs. The solid vertical black line indicates when the response was made. Hits are shown in teal, correct rejections (CRs) are shown in orange. Error bars reflect standard error of the mean. (A) Over the left central ROI, we find a significant interaction between response type and time interval (*p* < 0.001) driven by greater pre-response theta power for hits than CRs and numerically greater post-response theta power for CRs than hits. (B) Over the right central ROI, we find a significant interaction between response type and time interval (*p* = 0.027) driven by greater theta power for hits than CRs during the pre-response time interval.

Given the significant three-way interaction between response type, ROI, and time interval, we next performed follow-up post hoc ANOVAs over time separately for each ROI. We conducted two 2 × 15 rmANOVAs with factors of response type (hit, CR) and time interval (-500 to 1000 ms in fifteen 100 ms intervals). We report the results of this ANOVA in Table 3 and highlight the key findings here. For both ROIs (Fig. 3), we find a significant interaction between response type and time interval (LC: $F_{14,518}$ = 5.373, *p* < 0.0001, $\eta_{p}^{2}$ = 0.13; RC: $F_{14,518}$ = 3.791, *p* < 0.0001, $\eta_{p}^{2}$ = 0.09). This interaction was also significant when we separately considered strong and weak semantic items (strong, LC: $F_{14,518}$ = 3.297, *p* < 0.0001, $\eta_{p}^{2}$ = 0.08; strong, RC: $F_{14,518}$ = 2.796, *p* = 0.0005, $\eta_{p}^{2}$ = 0.07; weak, LC: $F_{14,518}$ = 4.232, *p* < 0.0001, $\eta_{p}^{2}$ = 0.10; weak, RC: $F_{14,518}$ = 2.041, *p* = 0.0137, $\eta_{p}^{2}$ = 0.05). These results demonstrate that theta power dissociations between hits and CRs vary across time interval, suggesting that differential processes may be engaged in a pre- versus post-response that distinguishes these response types.

**Table 3.** Analysis of variance results for response type (hit, CR) and time interval (-500 to 1000 ms in fifteen 100 ms intervals; pre-response, post-response) on theta power for the left and right central ROI.

Effect	*df*	-500 to 1000 ms interval						*df*	Pre vs. post response interval					
		Left central			Right central				Left central			Right central		
		*F*	*p*	$\eta_{p}^{2}$	*F*	*p*	$\eta_{p}^{2}$		*F*	*p*	$\eta_{p}^{2}$	*F*	*p*	$\eta_{p}^{2}$
Main effect of response type	(1,37)	0.382	0.54	0.01	0.007	0.934	0.0002	(1,37)	0.009	0.925	0.0002	0.871	0.357	0.02
Main effect of time interval	(14,518)	19.76	< **0.0001**	0.35	20.91	< **0.0001**	0.36	(1,37)	5.832	**0.0208**	0.14	0.708	0.406	0.02
Interaction of response type × time interval	(14,518)	5.373	< **0.0001**	0.13	3.791	< **0.0001**	0.09	(1,37)	16.18	**0.0003**	0.30	5.306	**0.027**	0.13

*Note*: Bold values indicate *p* < 0.05.

To specifically test for a pre- versus post-response dissociation in theta power for hits and CRs, we averaged signals within the 500 ms pre- and post-response time intervals (Fig. 3, insets). We conducted two 2 × 2 rmANOVAs, one for each ROI, with factors of response type (hit, CR) and time interval (pre-response, post-response). We report the results of this ANOVA in Table 3 and highlight the key findings here. In both ROIs, we find a significant interaction between response type and time interval. In LC, this interaction was driven by greater theta power for hits (M = 0.18, SD = 0.21) relative to CRs (M = 0.10, SD = 0.18) in the pre-response time interval ($t_{37}$ = 2.559, *p* = 0.0147, *d* = 0.4459) and numerically greater theta power for CRs (M = 0.05, SD = 0.25) relative to hits (M = -0.03, SD = 0.29) in the post-response time interval ($t_{37}$ = 2.021, *p* = 0.0506, *d* = 0.3019). In RC, this interaction was driven by numerically greater theta power for hits (M = 0.16, SD = 0.21) relative to CRs (M = 0.10, SD = 0.19) in the pre-response time interval ($t_{37}$ = 1.911, *p* = 0.0637, *d* = 0.295) and no difference in theta power for hits (M = 0.08, SD = 0.24) relative to CRs (M = 0.08, SD = 0.29) in the post-response time interval ($t_{37}$ = -0.1160, *p* = 0.9083, *d* = 0.015). We next tested the hemispheric specificity of this effect by directly comparing the post-response theta power dissociation across ROIs. We computed post-response difference scores (CRs minus hits) for each ROI. We find that the post-response theta power dissociation in LC (M = 0.081, SD = 0.25) does not significantly differ from the post-response theta power dissociation in RC (M = 0.004, SD = 0.21; $t_{37}$ = 1.605, *p* = 0.1171), indicating that the effect is not specific to the left hemisphere. Together, these results indicate that theta power is modulated by successful retrieval leading up to and following memory responses.

As the theta band encompasses multiple frequencies (4–8 Hz), we next tested the extent to which the center frequency of theta differed across response type and time interval. To specifically measure periodic signals, we fit the fitting oscillations and one over f (FOOOF) model to every test trial and extracted all identified center frequencies within the theta band prior to and following the response for the left and right central ROI. We compared the center frequency between hits and CRs pre- and post-response. We do not find any differences in center frequency for either ROI or time interval (LC, pre-response: $t_{37}$ = -0.0976, *p* = 0.9228; LC, post-response: $t_{37}$ = 0.2201, *p* = 0.8270; RC, pre-response: $t_{37}$ = -0.3237, *p* = 0.748; RC, post-response $t_{37}$ = -1.072, *p* = 0.2908). The center frequency for all conditions is generally around 5.6 Hz.

Prior work (Guan et al., 2023; Herweg et al., 2020) suggests that the pre-response theta power dissociation that we observe specifically reflects evidence accumulation or reinstatement that ultimately supports recollection. The post-response theta power effects may likewise reflect a feedback signal in response to recollected content. Due to the current task design, we cannot directly measure recollection and familiarity; however, we can leverage reaction time (RT) as a coarse assay of confidence. The general assumption is that compared to trials with slow RTs, trials with fast RTs reflect faster evidence accumulation (Mulder & van Maanen, 2013; Rollwage et al., 2020; Shenhav et al., 2018) and greater confidence (Ratcliff, 1978; Ratcliff & Starns, 2009; Weidemann & Kahana, 2016). To divide the trials based on RT, we calculated the median RT across all correct responses (hits and CRs) for each participant, and labeled hits as either “fast” (those below the median RT) or “slow” (those above the median RT). To the extent that fast hits reflect strongly remembered experiences or the degree of evidence accumulation, we should find differential theta power engagement pre- and post-response. Specifically, we should find greater pre-response theta power for fast hits compared to CRs, as there is no experience to remember or reinstate during a CR. To the extent that post-response theta power reflects a feedback signal based on reinstated content, we should find decreased theta power for fast hits compared to CRs.

**Fig. 4.**
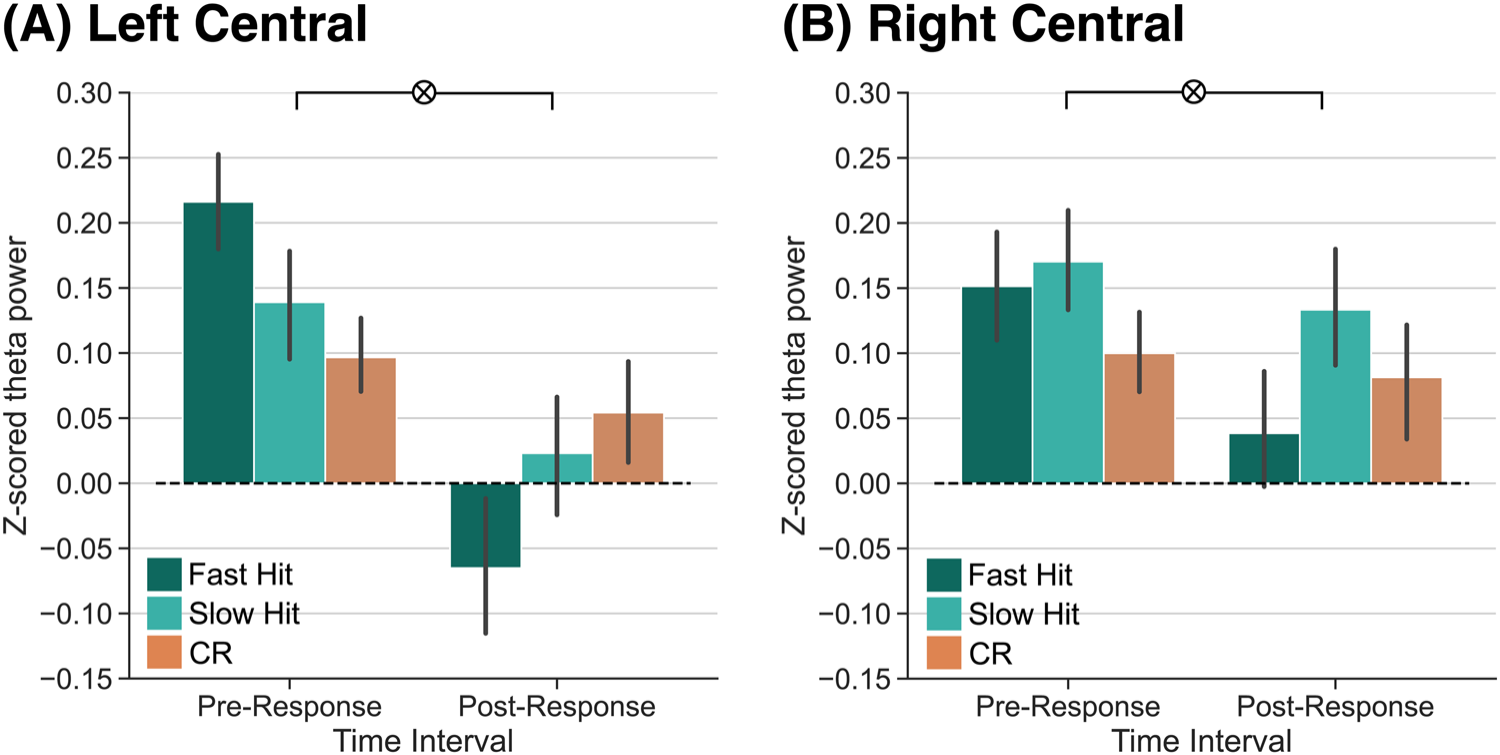
Theta power dissociations across fast hits, slow hits, and correct rejections preceding and following memory responses. Response-locked z-transformed theta power (4–8 Hz) for the left and right central ROIs. Fast hits are shown in dark teal, slow hits are shown in light teal, and correct rejections (CRs) are shown in orange. Error bars reflect standard error of the mean. (A) Over the left central ROI, we find a significant interaction between response type and time interval (*p* < 0.0001) driven by a significant pre-post interaction between fast hits and CRs (*p* < 0.0001). (B) Over the right central ROI, we find a significant interaction between response type and time interval (*p* = 0.0206) driven by a significant pre-post interaction between fast hits and CRs (*p* = 0.008).

To specifically test for a pre- versus post-response dissociation in theta power for fast hits, slow hits, and CRs, we averaged signals within the 500 ms pre- and post-response time intervals (Fig. 4). We conducted two 2 × 3 rmANOVAs, one for each ROI, with factors of time interval (pre-response, post-response) and response type (fast hit, slow hit, CR). We report the results of this ANOVA in Table 4 and highlight the key findings here. In LC, we find a significant interaction between response type and time interval. This interaction was driven by a significant interaction between both fast hits and CRs ($F_{1,37}$ = 24.56, *p* < 0.0001, $\eta_{p}^{2}$ = 0.40) and fast hits and slow hits ($F_{1,37}$ = 15.02, *p* = 0.0004, $\eta_{p}^{2}$ = 0.29). The interaction between slow hits and CRs was not significant ($F_{1,37}$ = 2.892, *p* = 0.0974, $\eta_{p}^{2}$ = 0.07). In the pre-response interval, theta power was significantly greater for fast hits (M = 0.22, SD = 0.23) relative to CRs (M = 0.10, SD = 0.18; $t_{37}$ = 3.080, *p =* 0.0039, *d* = 0.5789) and numerically greater for fast hits relative to slow hits (M = 0.14, SD = 0.25; $t_{37}$ = 1.908, *p =* 0.0642, *d* = 0.3256). In the post-response interval, theta power was significantly greater for CRs (M = 0.05, SD = 0.25) and slow hits (M = 0.02, SD = 0.28) relative to fast hits (M = -0.07, SD = 0.33; CRs vs. fast hits: $t_{37}$ = 2.554, *p* = 0.0149, *d* = 0.4161; slow hits vs. fast hits: $t_{37}$ = 2.606, *p =* 0.0131, *d* = 0.2918).

**Table 4.** Analysis of variance results for response type (fast hit, slow hit, CR) and time interval (pre-response, post-response) on theta power for the left and right central ROI.

Effect	*df*	Left central			Right central		
		*F*	*p*	$\eta_{p}^{2}$	*F*	*p*	$\eta_{p}^{2}$
Main effect of response type	(2,74)	0.019	0.981	0.0005	1.938	0.151	0.05
Main effect of time interval	(1,37)	7.459	**0.0096**	0.17	0.87	0.357	0.02
Interaction of response type × time interval	(2,74)	14.93	<**0.0001**	0.29	4.095	**0.0206**	0.10

*Note*: Bold values indicate *p* < 0.05.

In RC, we find a significant interaction between response type and time interval. This interaction was driven by a significant interaction between fast hits and CRs ($F_{1,37}$ = 7.885, *p* = 0.008, $\eta_{p}^{2}$ = 0.18), whereby theta power was numerically greater for fast hits (M = 0.15, SD = 0.26) relative to CRs (M = 0.10, SD = 0.19) in the pre-response time interval ($t_{37}$ = 1.274, *p* = 0.2107, *d* = 0.2278) and numerically greater for CRs (M = 0.08, SD = 0.29) relative to fast hits (M = 0.04, SD = 0.27) in the post-response time interval ($t_{37}$ = 0.9688, *p* = 0.3389, *d* = 0.1549). The interaction between slow hits and CRs was not significant ($F_{1,37}$ = 0.306, *p* = 0.583, $\eta_{p}^{2}$ = 0.008) nor was the interaction between fast hits and slow hits ($F_{1,37}$ = 4.038, *p* = 0.0518, $\eta_{p}^{2}$ = 0.010). Together, these results suggest that post-response theta power dissociations may reflect a positive feedback signal in response to strongly remembered experiences.

## 4 Discussion

The goal of the current study was to test the hypothesis that distinct processes occur prior to and following a memory response. We recorded scalp EEG while participants performed a recognition memory task and received no explicit feedback on their performance. Crucially, we focused our analyses on response-locked test-phase data to separate the processes occurring before and after a memory response. We show that reaction times (RTs) are faster for hits compared to correct rejections (CRs) and that a greater proportion of hits occur within 500–750 ms of stimulus onset compared to CRs. We replicate established findings (Nyhus & Curran, 2010) that preceding a memory response, theta power (4–8 Hz) in frontocentral electrodes is greater for hits compared to CRs. We show that these pre-response theta power dissociations “flip” in left central electrodes following the memory response. We find that this post-response “flip” is specific to hits committed quickly, potentially reflecting a positive feedback signal for strongly remembered experiences. Together, these findings suggest that there are potentially distinct memory and decision-making processes engaged preceding and following a memory response that are modulated by successful retrieval.

We find faster RTs for hits compared to CRs, replicating past findings (Uncapher et al., 2015; Weidemann & Kahana, 2016). Faster RTs for hits may be driven by greater memory strength (Verde & Rotello, 2007; Wixted, 2007) and/or greater contextual reinstatement (Gordon et al., 2014; Hanczakowski et al., 2015). However, these RT differences indicate that traditional hit versus CR comparisons of stimulus-locked data may capture both pre- and post-response related processes. We specifically find that a larger proportion of hits occur within 500–750 ms of stimulus onset compared to CRs meaning that if participants evaluate or update a representation after responding, and they respond at different times for hits relative to CRs, estimates of neural signals within this time window may reveal differences in post-hit evaluation processes following successful retrieval versus memory/retrieval-related processes related to correctly rejecting lures. Thus, our results highlight the importance of utilizing response-locked data to investigate the distinct processes leading up to and following a memory response.

Consistent with prior EEG work (Burgess & Gruzelier, 1997; Düzel et al., 2003; Klimesch et al., 1997, 2000; Nyhus & Curran, 2010), we find greater pre-response theta power for hits than CRs. EEG power in the theta frequency band has been proposed to coordinate cortical areas, supporting the ability to encode and retrieve contextual information about time and space that is central to episodic memory (Hasselmo & Stern, 2014). Successful memory retrieval depends on the access of an internal representation of a past experience. This access may take the form of reinstatement, wherein encoded content is reconstructed during retrieval (Danker & Anderson, 2010), which may specifically be supported by coordination between cortical areas (Herweg et al., 2020). Our findings are generally consistent with the drift diffusion model (Ratcliff & McKoon, 2008; Ratcliff et al., 2016) whereby the pre-response theta power dissociations may reflect evidence accumulation. Insofar as pre-response theta power reflects reinstatement of a past experience (Guan et al., 2023; Kota et al., 2020), our finding of generally lower pre-response theta power for CRs is consistent with the evidence accumulation account as there is no experience to reinstate during a CR. Although we did not fit the drift diffusion model to the neural data, we would predict that greater theta power preceding a response would be associated with a higher or faster drift rate, the model parameter that reflects the strength of the rate of evidence accumulation (Ratcliff et al., 2016). This interpretation is consistent with our finding of robust pre-response theta power dissociations specifically between slow hits and fast hits. Fast RTs are thought to reflect rapid evidence accumulation (Mulder & van Maanen, 2013; Rollwage et al., 2020; Shenhav et al., 2018), thus greater pre-response theta power for fast hits compared to slow hits may reflect faster evidence accumulation for strongly remembered experiences.

Following a memory response, we find a “flip” in theta power over the left central ROI, such that theta power is greater for CRs than hits. Participants did not receive feedback indicating that they were correct suggesting that the dissociation in theta power may occur in response to intrinsic feedback signals specifically following successful retrieval. Although prior work has demonstrated that post-retrieval monitoring processes are engaged following memory retrieval (Hill et al., 2021; Rugg et al., 2003), the majority of these findings reflect signals that precede a behavioral response. Our interpretation is that the post-response theta power dissociation between hits and CRs may reflect a feedback signal. This interpretation is consistent with work from the cognitive control literature showing that theta power increases for incorrect compared to correct responses and following negative relative to positive outcomes (Cavanagh & Frank, 2014; Cavanagh et al., 2010). Prior work has proposed that frontal midline theta (FMT) is associated with reward processing—specifically that the FMT is larger following negative feedback or monetary loss (Cohen et al., 2007; Marco-Pallarés et al., 2008)—and is a mechanism for communication between brain regions (Glazer et al., 2018). As both hits and CRs constitute correct responses, we may have anticipated decreased post-response theta power for both response types. However, only hits reflect successful retrieval. Thus, the post-response decrease in theta power for hits may reflect positive feedback specifically in response to successful retrieval.

We find that the pre- versus post-response dissociation in theta power is specific to fast hits. To the extent that fast hits reflect highly confident responses (Ratcliff, 1978; Ratcliff & Starns, 2009; Weidemann & Kahana, 2016), the post-response theta power decrease for fast hits may reflect a positive feedback signal. Neuro-imaging work has repeatedly shown that reward-related regions (e.g., striatum) are more active during hits compared to CRs in the absence of explicit reward (Achim & Lepage, 2005; Clos et al., 2015; de Zubicaray et al., 2005; Fliessbach et al., 2006; Henson, et al., 2005; Schwarze et al., 2013; Spaniol et al., 2009). As both responses are correct, the dissociation in striatal activity for hits compared to CRs suggests that the signal change is not driven by overall accuracy, but rather is a response to successful retrieval and indicates that successful retrieval may be intrinsically rewarding (Satterthwaite et al., 2012; Speer et al., 2014). Taken together, the post-response theta dissociation that we observe in the current study may represent a positive feedback signal in response to successful retrieval, though a direct test of this account is necessary to support this claim. The direct investigation of post-response test-phase feedback signals presents an exciting avenue for future work.

Our broad interpretation of the present findings is that the post-response theta power decrease reflects the last process in a cascade of processes that support “remembering,” generally construed. An individual begins remembering by perceptually processing an externally presented stimulus and then engages in a memory search, internal attention, evidence accumulation, and/or matching process in an attempt to access a stored representation (Kahana, 2012; Polyn & Kahana, 2007; Ratcliff et al., 2016; Smith & Long, 2024)—these two processes need not be serial and instead an individual may iterate between them. If these processes are successful, an item—and possibly its context or other associated information—may be reinstated, as in Tulving’s original proposal of ecphory (Tulving, 1983). Reinstatement is followed by a monitoring process in which the accessed representation is evaluated (Cruse & Wilding, 2009). A decision is then made in tandem with commission of a behavioral response. Finally, a decision- making post-response evaluative or representation updating process is engaged. Our interpretation is that the frontocentral post-response theta dissociation reflects this final decision making step. Given the limitations in estimating reinstatement of verbal, as opposed to visual, stimuli (Brunec et al., 2020), future work is needed to directly test this account and the timing and relationship between reinstatement and post-decision signals.

A critical open question is how the observed RT distributions and pre- versus post-response theta dissociations relate to the established processes of recollection and familiarity. Recollection is the retrieval of contextual details and familiarity is memory strength without detailed retrieval (Addante et al., 2012; Diana et al., 2005; Gimbel & Brewer, 2011; Yonelinas, 2001a). There is mixed evidence as regards RTs for recollection and familiarity, with some evidence that recollection responses are faster than familiarity responses (Diana et al., 2005; Gimbel & Brewer, 2011; Herweg et al., 2016) and some evidence that recollection responses are slower than familiarity responses (Atkinson & Juola, 1974; Besson et al., 2012; Jacoby, 1991). Due to our task design, we cannot disambiguate recollection from familiarity based responses, but we can leverage RT as a coarse assay of confidence. Our assumption is that compared to slow hits, fast hits reflect higher confident responses (Ratcliff, 1978; Ratcliff & Starns, 2009; Weidemann & Kahana, 2016). High confident responses may be supported by the recollection of contextual details (Yonelinas, 2001a, 2001b), in which case elevated pre-response theta power may reflect the reinstatement of contextual details during fast hits. Likewise, the decreased post-response theta power following fast hits may reflect a positive feedback signal in response specifically to recollected experiences. Future work will be needed to directly test this possibility.

Although we did not anticipate hemispheric differences, we consistently found influences of ROI on theta power. Overall, the effects that we observe are numerically stronger in the left central, relative to right central, ROI, although a direct test of the post-response theta dissociation for left versus right ROIs indicated that the effect is not specific to the left hemisphere. It is unlikely that motor responses contributed to the observed hemispheric effects given that responses were counterbalanced across participants and typically motor movements engage higher frequency bands (e.g., Crone et al., 1998). The hemispheric asymmetry may be driven by the stimuli used and/or intrinsic hemispheric connections (D. Wang et al., 2014). We used visually presented words in the current study and verbal stimuli are well known to recruit the left hemisphere (de Zubicaray et al., 2011; Ries et al., 2016; Price, 2012; Vigneau et al., 2011), including during memory tasks (Kelley et al., 1998; Kim, 2011). Investigation of resting state data has shown that cortical networks have intrinsic within-hemisphere connections which may enable control over the specific functions or processes that are engaged (D. Wang et al., 2014). Future work will be needed to probe both the hemispheric asymmetry of this effect as well as the overall spatial specificity, given that we chose to focus exclusively on frontocentral regions due to the convergence of memory and decision-making literature on this area. Although a region-frequency focused investigation is ideal for hypothesis testing (Cohen, 2014a), it does mean that other important neural substrates may be engaged following successful retrieval that we were not positioned to identify. Indeed, other areas are known to also support memory retrieval (e.g., right frontal, Evans et al., 2015; left parietal, Jacobs et al., 2006) which may also show post-response dissociations. Our findings generally show a frontocentral theta pattern consistent with pre-response memory-related processing and post-response decision-making-related processing.

Together, our findings suggest that distinct processes occur prior to and following a memory response and, in particular, that decision-making processes may follow successful retrieval. A direction for future research will be to directly investigate the extent to which the post-response theta dissociation reflects a feedback signal. More broadly, we contribute to a growing body of literature characterizing the role of theta activity in successful memory retrieval.

## Data Availability

The raw, de-identified data and the associated experimental and analysis codes used in this study can be accessed via the Long Term Memory Lab Website (https://longtermmemorylab.com).
